# Computational Design of Antiperovskite Solid Electrolytes

**DOI:** 10.1021/acs.jpcc.3c04953

**Published:** 2023-09-12

**Authors:** Ana C. C. Dutra, James A. Dawson

**Affiliations:** † Chemistry − School of Natural and Environmental Sciences, Newcastle University, Newcastle upon Tyne NE1 7RU, U.K.; ‡ Centre for Energy, Newcastle University, Newcastle upon Tyne NE1 7RU, U.K.; § The Faraday Institution, Didcot OX11 0RA, U.K.

## Abstract

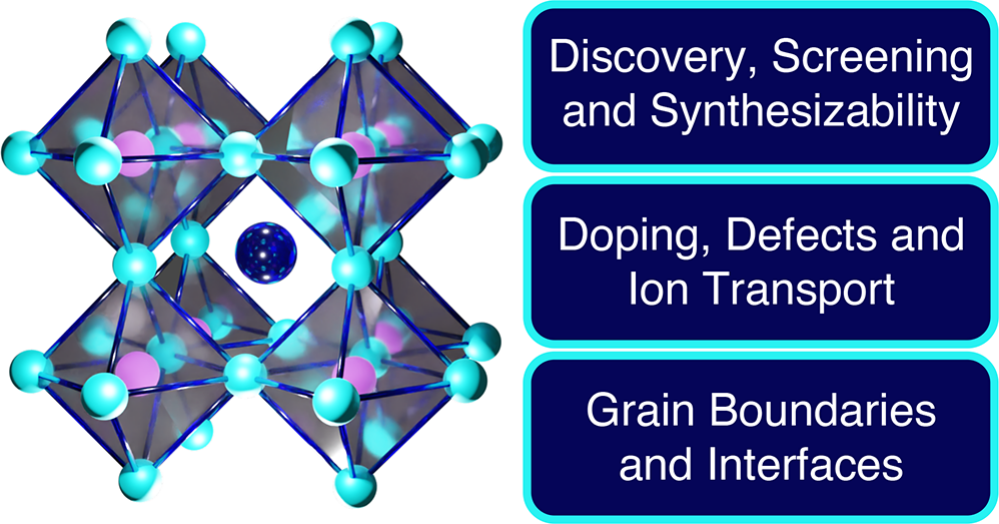

In the face of the current climate emergency and the performance, safety, and cost limitations current state-of-art Li-ion batteries present, solid-state batteries are widely anticipated to revolutionize energy storage. The heart of this technology lies in the substitution of liquid electrolytes with solid counterparts, resulting in potential critical advantages, such as higher energy density and safety profiles. In recent years, antiperovskites have become one of the most studied solid electrolyte families for solid-state battery applications as a result of their salient advantages, which include high ionic conductivity, structural versatility, low cost, and stability against metal anodes. This Review highlights the latest progress in the computational design of Li- and Na-based antiperovskite solid electrolytes, focusing on critical topics for their development, including high-throughput screening for novel compositions, synthesizability, doping, ion transport mechanisms, grain boundaries, and electrolyte–electrode interfaces. Moreover, we discuss the remaining challenges facing these materials and provide our perspective on their possible future advances and applications.

## INTRODUCTION

The search for sustainable energy storage technologies to power the electrification of transport and the large-scale storage of intermittently generated renewable energy has become crucial as the world faces a climate emergency and attempts to migrate to a net-zero economy. Despite powering the revolution in portable electronics, current Li-ion batteries face critical performance, cost, and safety limitations that may limit their ability to power future leading-edge applications.^1−5^

In this context, solid-state batteries (SSBs) are currently capturing significant interest as a next-generation technology endowed with a plethora of potential transformative advantages compared to traditional Li-ion batteries, including improved energy density and safety.^2,3,6−11^ In contrast to commercial rechargeable batteries, which are powered by liquid electrolytes, SSBs utilize solid electrolytes to provide fast ionic conduction between the battery electrodes. As a result, the heart of this technology and the key to its future success lies in the development of solid electrolytes with lower costs, suitable mechanical properties, and high ionic conductivity, stability, synthesizability, scalability, and electrode compatibility.

Consequently, research featuring the discovery and design of such powerful electrolytes has soared in recent years. Although promising and competitive materials in terms of conductivity have been identified and developed, including sulfides (e.g., Li_10_GeP_2_S_12_),^12−14^ oxides (e.g., Li_7_La_3_Zr_2_O_12_),^15,16^ and halides (e.g., Li_3_InCl_6_),^17,18^ the search for a solid electrolyte material that displays a suitable ensemble of qualities that could power the widespread and large-scale use of SSBs is still ongoing.

**Figure 1:**
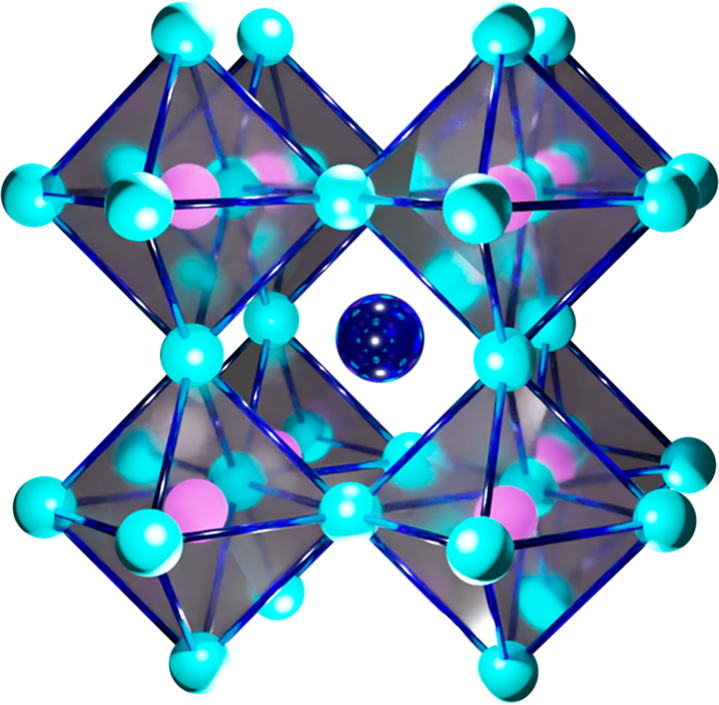
Cubic structure (*Pm*3̅m) of an antiperovskite with the general formula X_3_BA. The A and B sites (dark blue and pink, respectively) are occupied by anions, while a cation occupies the X site (light blue). The B site is octahedrally coordinated to six X-site cations, and the A-site anions are cuboctahedrally coordinated to 12 nearest-neighbor cations.

In recent years, antiperovskites, with a typical formula of X_3_OA (X = Li or Na; A = Cl, Br, I or a mixture of halides), have risen to become one of the most promising solid electrolyte families under consideration for SSB applications. This promise is based on their excellent features, such as high ionic conductivity (>10^–3^ S cm^–1^), structural versatility, wide electrochemical windows, low cost, flexible crystal structure, and stability against Li metal,^2,19−22^ an ensemble of advantages rarely seen concomitantly in other solid electrolyte candidates. The cubic antiperovskite structure is displayed in Figure 1.

Nevertheless, the application of antiperovskites has been hindered by several pressing issues, including misconceptions regarding their stability and synthesizability and their strongly hydroscopic nature.^2^ In addition, there are still fundamental challenges inherent to SSBs, which affect most solid electrolyte candidates and remain unsolved, including lithium dendrite growth, electrochemical stability, large-scale synthesis and interfacial resistance.^2,3^

Computational modeling plays an important role in tackling these critical challenges, either by predicting novel high-performance solid electrolyte compositions or by providing a deeper understanding of the barriers preventing SSBs from being applied at larger scales.^2,6,23,24^ In the context of antiperovskites, atomistic simulations based on density functional theory (DFT), *ab initio* molecular dynamics (AIMD), and force-field-based molecular dynamics (MD) have been fundamental in understanding and tailoring a wide array of important phenomena and properties, including ionic and electronic conductivity, diffusion mechanisms, stability, defects and doping, and interfacial resistance and compatibility. Furthermore, machine learning (ML) and high-throughput approaches have proven to be powerful for identifying and screening promising antiperovskite compositions.^25,26^ Computational modeling has therefore become a vital tool in complementing and assisting the experimental synthesis and characterization of antiperovskites, as well providing key insights that cannot be achieved experimentally.

In this Review, we highlight some of the latest advances in the computational design of Li- and Na-based antiperovskite solid electrolytes, focusing on critical topics for their further development and large-scale implementation. Specifically, we offer a timely review of the most recent progress made in the high-throughput screening of novel antiperovskite compositions and synthesizability predictions, both key to the discovery of efficient and stable solid electrolytes. We then discuss recent computational reports on ion transport mechanisms, defect chemistry, and doping in antiperovskites. In light of their role as significant barriers to the development of SSBs, we also feature studies on surfaces, grain boundaries, and electrode–electrolyte interfaces. Finally, we summarize the progress that has been achieved to date and the remaining critical challenges as well as provide our opinions on the exciting future for this ever-evolving field.

## DISCOVERY, SCREENING, AND SYNTHESIZABILITY

Considering the fundamental role solid electrolytes play in delivering fast ionic conduction in SSBs, the success of future SSB implementations lies heavily in the discovery of efficient, stable, and synthesizable solid electrolytes. Atomistic simulations can contribute greatly to this task by providing powerful predictions that can help guide future experimental directions, resulting in more successful synthesis attempts with improved time and resource utilization.

Discovery and compositional screening studies of antiperovskites have provided fundamental information regarding how different compositions and chemistries can affect the potential performance of these materials as solid electrolytes. Such investigations are particularly interesting when comparing Li-ion-based systems to different chemistries such as Na-ion-based antiperovskites. Due to the wide availability and lower costs related to sodium, these are becoming increasingly appealing for battery technologies.

**Figure 2:**
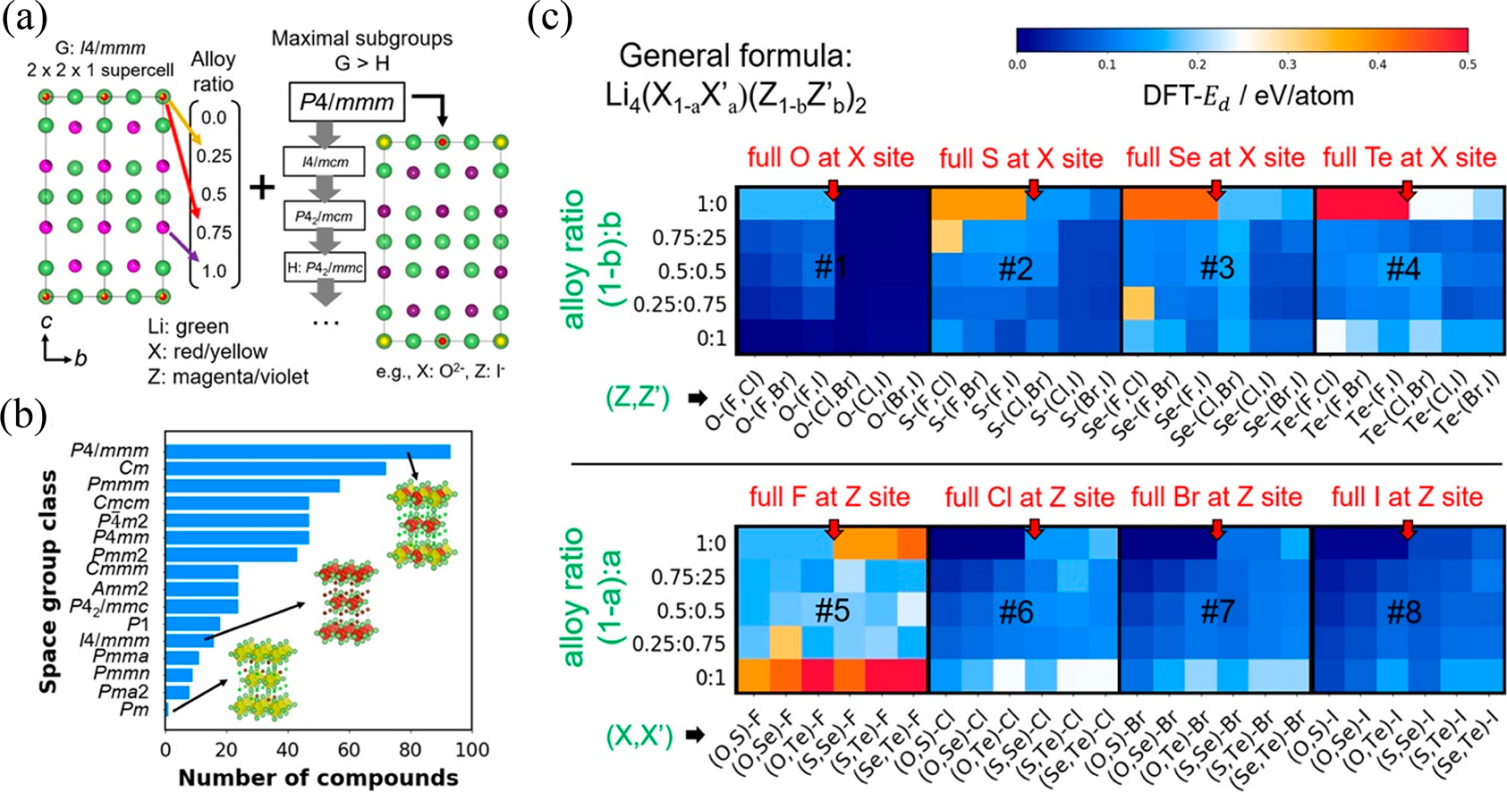
(a) Schematic illustration for the generation of tetragonal Ruddlesden–Popper antiperovskites. (b) Histogram plot of in silico generated antiperovskites with their space groups from the procedure in (a). (c) Thermodynamic stability map for in silico generated antiperovskites with one-element full occupancy at one anion site (red), while the other anion site is varied (green). Inset numbers indicate the subplot number (with a #) for each chemical substitution case. The color map represents the DFT decomposition energy (*E*_*d*_) values with *E*_*d*_ < 0.1 eV/atom set as the phase stability criterion. Vertical axes (red and green) show substitution ratios corresponding to X/Z combinations described in the horizontal axes. Element combinations (X,X′–Z,Z′) are shown inside parentheses. Reproduced with permission from ref (29). Copyright 2021 American Chemical Society.

One example is our previous investigation of a wide range of Li_3–*x*_Na_*x*_OCl_1–*y*_Br_*y*_ compositions,^27^ which showed the effect that halide-ion mixing has on the conductivity displayed by such systems, the low conductivities and high activation energies for mixed Li/Na systems, and the more prominent Li-ion conductivity when compared to Na-ion conductivity. Li- and Na-based antiperovskites with Ruddlesden–Popper structures have also received significant attention in recent years. Interestingly, Yu et al.^28^ revealed that Li/Na mixing in antiperovskites with the general formulas of Na_4–*c*_Li_*c*_AX_4_ (A = O and/or S; X = I and/or Cl) could lead to a promising solid electrolyte candidate, Na_3_LiS_0.5_O_0.5_I_2_, which displayed a very low activation energy of 0.12 eV, a room temperature (RT) ionic conductivity of 6.3 mS cm^–1^, and both thermo- and electrochemical compatibility with sodium metal anodes. The most promising candidate based solely on sodium reported by the authors was Na_4_S_0.5_O_0.5_I_2_, which possessed a RT ionic conductivity of 0.347 mS cm^–1^ and an activation energy of 0.23 eV. More recently, Jalem et al.^29^ explored a material space of more than 500 Li-rich antiperovskites with the *n* = 1 Ruddlesden–Popper tetragonal structure via DFT, MD, and phonon calculations to find promising solid electrolyte candidates based on critical features toward battery applications such as thermal and electrochemical stability, Li-ion transport and surface properties (Figure 2). The authors predicted that 167 compounds are thermodynamically (meta)stable and highlighted 20 novel compounds with a decomposition energy below 0.05 eV/atom as well as revealing a low likelihood for lithium dendrite formation in these compounds. Such results illustrate the potential of antiperovskite solid electrolytes with Ruddlesden–Popper structures.

Li- and Na-rich antiperovskites based on cluster ions (e.g., BH_4_, AlH_4_, BF_4_, and BCl_4_) have also shown promising results. In a DFT study, Fang and Jena^21^ explored a set of Li-rich antiperovskites based on cluster ions (Li_3_O^+^/Li_3_S^+^ and BH_4_^–^/AlH_4_^–^/BF_4_^–^), and their results suggested that cluster ions can produce larger band gaps and channel sizes in antiperovskite structures, generating a larger environment for Li ions to diffuse. A larger channel size also generates low-energy phonon modes, which facilitate Li-ion migration by constantly changing the potential surface across the material. Structures including Li_3_SBF_4_, Li_3_S(BF_4_)_0.5_Cl_0.5_, Li_3_O(BH_4_), and Li_3_O(BH_4_)_0.5_Cl_0.5_ were reported as promising solid electrolytes^21,30^ with RT conductivities of 10^–2^, 10^–1^, 10^–4^, and >10^–3^ S cm^–1^, respectively. Na-rich-cluster-based antiperovskites were also studied by Fang and Jena^31^ in a design work that reported Na_3_S(BCl_4_) and Na_3_S(BCl_4_)_0.5_I_0.5_ as promising solid electrolytes, displaying RT ionic conductivities of >10^–3^ S cm^–1^, low activation energies (<0.2 eV), large bandgaps, and suitable mechanical properties. Orientational shifts in the tetrahedral cluster ion were also reported to reduce the ionic migration barrier and the preference for Na ions to remain at their lattice sites.^31^

More recently, Xu et al.^32^ used DFT calculations to explore stability, electronic properties, elastic constants, and Na-ion migration in cluster-based antiperovskites with the formula Na_3_SA (A = AlF_4_, ClO_4_, ICl_4_, and IO_4_). The results achieved revealed that cluster substitutions at the A site can lead to larger band gaps and higher ion transport in the selected antiperovskites when compared to their counterparts that contain halides on their A site. MD simulations suggested that the improved ion transport arises from the rotation of cluster ions and the large volume created inside the crystal structure. However, it is noteworthy that cluster substitutions can also lead to lattice distortions that can lower the stability of the material. The authors determined Na_3_SAlF_4_ to be the best candidate tested, with an activation energy of 0.19 eV, a stable structure, an ionic conductivity of 6.55 × 10^–2^ S cm^–1^ at RT, and a migration barrier of 0.46 eV.

Although the above atomistic studies provide critical information for the fundamental understanding of antiperovskite solid electrolytes, given the great structural and chemical diversity they present, high-throughput screening and ML methods are also essential for their timely further development.

The first high-throughput screening focused on antiperovskites was carried out by Singh et al. in 2018,^33^ where DFT and phonon calculations were used to evaluate the thermodynamical, mechanical, and dynamical stability of 630 cubic magnetic antiperovskites. Although this study did not explore antiperovskites as solid electrolytes, it identified 11 novel antiperovskite compositions, thereby illustrating the potential of such an approach. Concurrently, ML has been used for the binary classification of crystal compounds as perovskites or nonperovskites,^34^ with artificial neural networks successfully used to classify compositions as cubic antiperovskites and identify those that display octahedral rotation.

**Figure 3:**
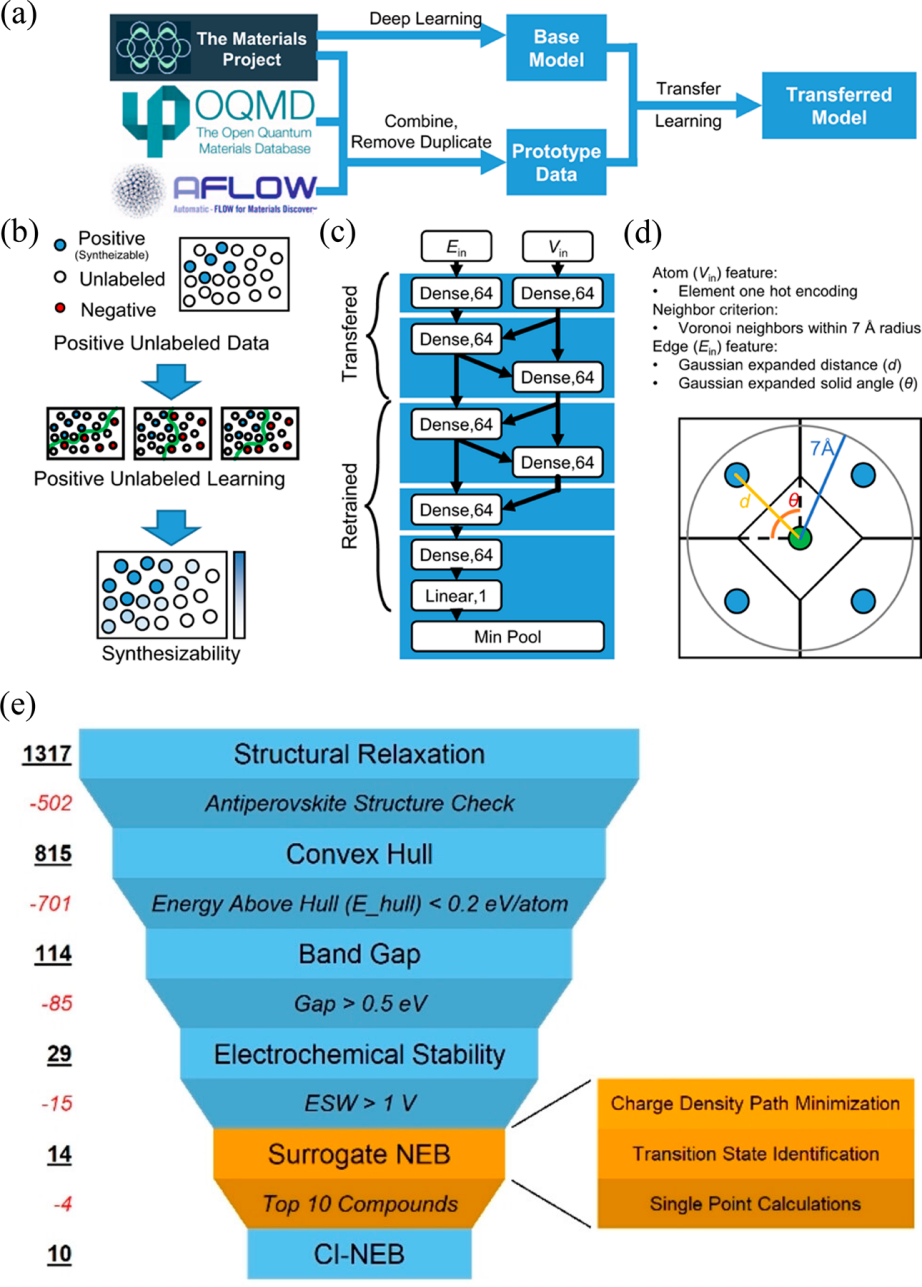
(a) Domain-specific transfer learning workflow used to retrieve perovskite structures. The model is first trained with the Materials Project database and then retrained with the perovskite-only data extracted from the three databases. (b) Overview of positive and unlabeled learning procedure. (c) Graph neural network architecture where *E*_in_ and *V*_in_ are the atom and edge features, respectively. Dense indicates the linear multiplication followed by the softplus activation layer, and Linear indicates linear multiplication. The number next to the operation indicates the output feature dimension. Min Pool indicates minimum pooling followed by sigmoid activation. (d) Crystal representation with atoms and edges converted to mathematical representation via featurization. (a–d) Reproduced with permission from ref (25). Copyright 2022 The Authors. (e) A visualization of the steps in the workflow used to screen antiperovskite candidates, along with the filtering criteria used to lower the total computational cost of the screening. The underscored and italic numbers represent the number of candidates present at each step and the number lost to each criterion, respectively. The inset illustrates the steps of the surrogate nudged elastic band (NEB) method. Reproduced with permission from ref (26). Copyright 2023 Wiley-VCH.

More recently, ML has also been used as a powerful tool for predicting synthesizability in antiperovskites. As seen with the development of many others prediction methods for antiperovskites, the progress in ML for synthesizability predictions also started with efforts focused primarily on common perovskites.^25,35−40^ Unfortunately, due to their focus on metal oxide perovskites or reliance on the Shannon ionic radii database, many of these models could not successfully be used for antiperovskite predictions.^25^ However, this scenario changed recently, with Gu et al. reporting a general graph neural network model capable of assessing the synthesizability of both common perovskites and antiperovskites based on structural and thermodynamic data taken from several well-known materials databases (Figure 3(a)–(d)).^25^ The model predicted 327 virtual antiperovskites to be synthesizable, including Na-rich antiperovskites considered as solid electrolytes, namely, Na_3_OBr and Na_3_OF. The study, however, also stated the importance of considering their findings from ML in the context of thermodynamic metrics to produce more accurate predictions.

ML methods have also proved to be useful in accelerating the evaluation of the kinetic properties of antiperovskites. One of the most critical bottlenecks related to the computational investigation of ion diffusion barriers is the use of time-consuming nudged elastic band (NEB) calculations. As NEB simulations demand a supercell approach and several DFT simulations to calculate the energy and forces, the computational cost for NEB-based simulations can be significant, thereby rendering NEB methods unsuitable for large screening studies. To accelerate NEB simulations, different methods have been proposed in recent years, such as the use of crystal symmetries and ML for the identification of transition states.^41,42^ In particular, Sjølin et al.^26^ developed a multitarget multifidelity workflow that replaces the need for expensive NEB simulations, as schematically summarized in Figure 3(e). The authors overcame the NEB bottleneck by using a surrogate model capable of identifying the transition-state structure during ionic diffusion and developed the workflow to systematically assess thermodynamic and electrochemical stability and electronic and ionic conductivity. Upon application of the workflow on the chemical space of antiperovskites, Sjølin et al. identified 14 solid electrolyte candidates, all of which had already been identified by other experimental and computational studies. Although such results illustrate the difficulty in discovering novel antiperovskite structures, they show how unprecedented advances in the field can be achieved by employing surrogate model-assisted workflows, as conclusions that took traditional studies years to uncover can be rapidly obtained by using such methods. This work also opens the interesting question of whether similar results would be expected for space groups with distortions (e.g., octahedral tilting) and for low-dimensional-networked antiperovskites.^6,43^

One of the many challenges involved in predicting and confirming a material as a promising solid electrolyte is that its properties can very often compromise or contradict each another. For example, in the recent study of Gu et al.^25^ that used graph neural networks to predict the synthesizability of perovskites, eight antiperovskite systems, namely, Li_3_CHg, Li_3_BeIr, Li_3_CBe, Li_3_ClSr, Li_3_SPb, Li_3_FK, Li_3_BeTi, and Li_3_PMn, simultaneously displayed good synthesizability scores but low thermodynamic stabilities, especially when compared to those computed for Li_3_OBr and Li_3_OCl. A similar scenario was also observed in our recent work,^6^ where the zero-dimensional antiperovskite Li_6_OBr_4_ displayed an exceptionally high Li-ion diffusion coefficient of 6.08 × 10^–9^ cm^2^ s^–1^ at 300 K but was unstable at higher temperatures and has yet to be experimentally realized. Such examples illustrate the importance of establishing a multitargeted search for novel solid electrolytes that focuses concomitantly on multiple properties (e.g., ionic conductivity, thermodynamic stability, electrochemical stability, and synthesizability) to avoid incomplete analyses that can lead to premature promising status declarations.

In this context, studies that explore the impact different chemical, mechanical, and structural features have on ion mobility, stability, and synthesizability and investigate possible intrinsic correlations between such features are vital for the further development of solid electrolyte candidates.

Important insights regarding the connections between lattice distortions, stability, and ionic mobility in antiperovskites were obtained by Kim and Siegel^24^ in a work that assessed 24 antiperovskites with the general formula X_3_BA (X = Li or Na; B = O, S or Se; A = F, Cl, Br or I). The authors introduced lattice distortions (e.g., tilting/rotation of polyhedral building blocks, bond length variations, and symmetry lowering/shifting) via isovalent substitutions and used DFT simulations to calculate the energy barriers for ion migration pathways, assuming both vacancy and interstitial mechanisms. A strong correlation was found between the magnitude of lattice distortions and energy barriers, with larger distortions providing lower energy barriers for ion migration regardless of the preferred migration mechanism. This work also revealed a correlation between the degree of lattice distortion and thermodynamical stability where higher lattice distortions are accompanied by lower stabilities. These results made evident that promising solid electrolyte candidates need an appropriate balance between mobility and stability. The authors suggested Na_3_SI as a balanced candidate in terms of stability and ionic conductivity. Na_3_SeF, Na_3_SeI, Li_3_SI, and Na_3_SF were also highlighted as being worthy of future experimental exploration.

More recently, in an experimental–computational work, Kim et al.^44^ used DFT calculations alongside the quasi-harmonic approximation to investigate the thermal stability and synthesizability of metastable antiperovskites with the general formula X_3_BA (X = Li, Na, or K; B = O, S, or Se; A = F, Cl, Br, or I). In this study, a linear correlation between the degree of lattice distortion (i.e., the tilting of the alkali metal octahedra and consequent perturbations to bond lengths and angles) and the stabilization temperature was found, indicating that antiperovskites endowed with the highest ionic mobility will usually demand the highest synthesis temperatures. This data guided experimental efforts that successfully resulted in the synthesis of Na_3_OA (A = Cl, Br, or I) and Li_2_OHA (A = Cl or Br) and showed overall good agreement with the computational predictions, indicating that the 0 K decomposition energy of a solid electrolyte can be a suitable descriptor for assessing the complexity and likelihood of its synthesis.

Relationships between structural features, stability, and ionic diffusion were also reported in our recent work^6^ that explored low-dimensional antiperovskites with the general formula Li_*x*_OA_*x*-2_ (A = Cl or Br; *x* = 3–6), as introduced previously by Lu et al.^43^ Through force-field-based MD simulations, we revealed a strong correlation between ionic diffusion and dimensionality in these structures, namely, increasing Li-ion diffusion and decreasing activation energy with reduced dimensionality, as well as instability at temperatures over 300 K for materials with dimensionalities lower than two, highlighting the difficulty in synthesizing such compounds.

As a crucial step when designing and assessing new antiperovskite materials, accurately predicting their synthesizability has proved to be challenging. It has been discussed widely in the literature^2,37,45^ how the Goldschmidt tolerance factor, a traditional indicator of stability, is not a suitable descriptor for antiperovskite materials, especially those containing heavy halides, such as Cl, Br, or I, and cluster ions, such as BH_4_, BF_4_, or NO_2_. One of the reasons for such unsuitability is the use of Shannon radii values, which can potentially contribute to a nonrepresentative description of the ion environment and, consequently, inaccuracies when using the Goldschmidt factor. Attempts to refine the radii for such calculations have been reported^2,37,45^ and a modified Goldschmidt tolerance factor formula recently proposed by Jalem et al.^29^ proved to be successful as a descriptor for thermodynamic stability and band gap energy for antiperovskites with the *n* = 1 Ruddlesden–Popper tetragonal structure.

## DOPING, DEFECTS, AND ION TRANSPORT MECHANISMS

**Figure 4:**
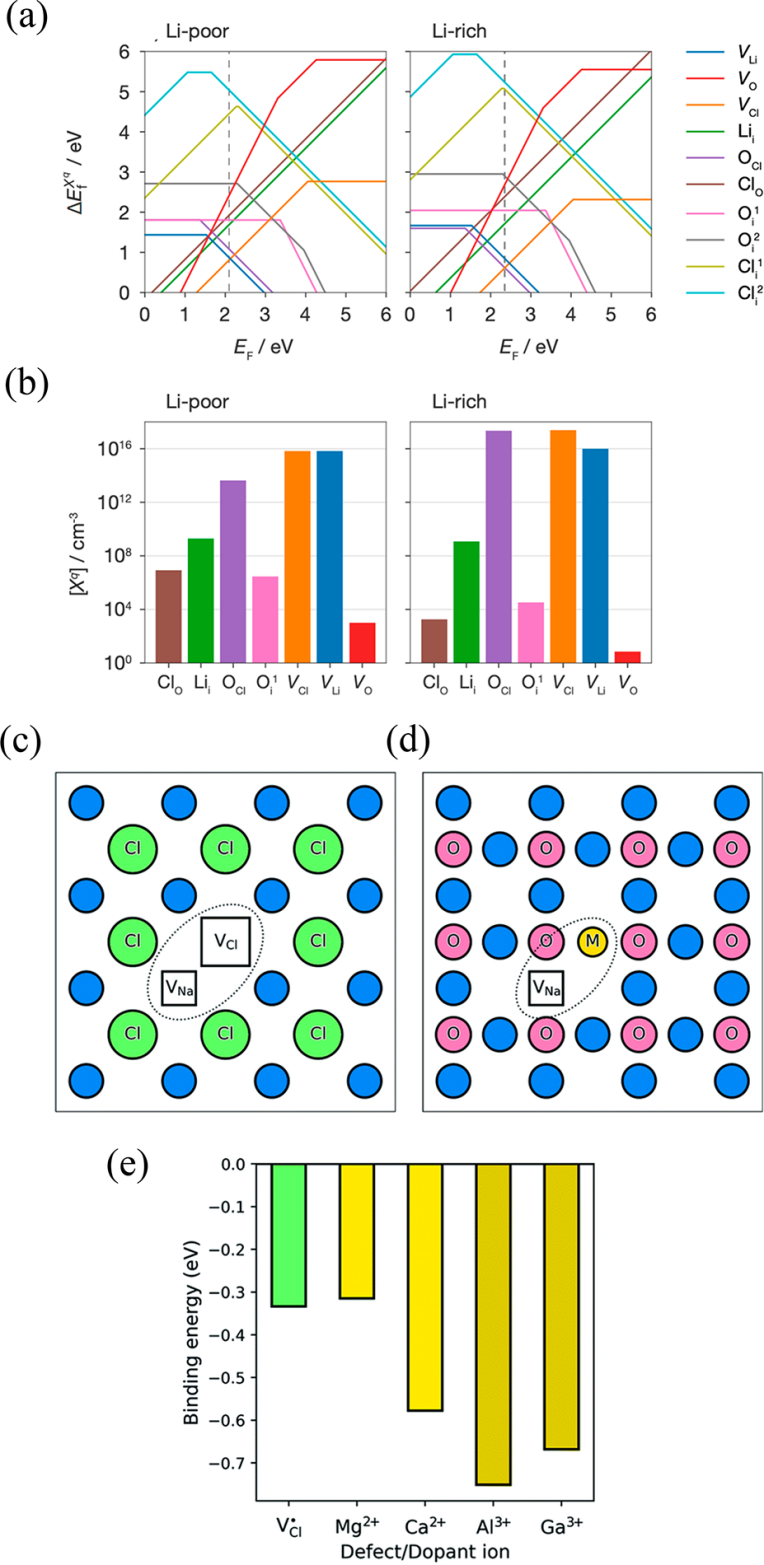
(a) Formation energies and (b) concentrations for defects in Li_3_OCl under Li-poor and Li-rich synthesis conditions at 360 °C. The dashed line marked on the transition level diagrams is the position of the self-consistently determined Fermi energy, and defect charge states are given by the gradient. Adapted from ref (46) under CC-BY license terms. Copyright 2023 The Authors. Defect clustering in Na_3_OCl: (c) undoped structure with sodium and chloride vacancies; (d) doped structures with a sodium vacancy and a dopant ion; (e) binding energies for defect/dopant clusters. Reproduced from ref (49) under a CC BY 3.0 license.

Research into understanding how different doping approaches can impact ionic conduction in solid electrolyte candidates is fundamental to the development of solid-state batteries. In this context, Squires et al.^46^ used DFT calculations to investigate the defect chemistry of Li_3_OCl and the impact that supervalent and subvalent doping has on its native defect concentrations (e.g., vacancy, interstitial, and anion antisite defects) and ionic conductivity. As shown in Figure 4(a),(b), the authors found that lithium and chlorine vacancies were the dominant negatively and positively charged defect species in undoped Li_3_OCl, respectively, under Li-poor conditions. Under Li-rich conditions, the dominant disorder types found were the oxygen–chlorine antisite and lithium vacancy, respectively. The identification of this antisite as one of the preferred disorder types shows the importance of investigating beyond Schottky and Frenkel-type defects when analyzing the defect chemistry of antiperovskites. Importantly, lithium vacancies were predicted to be present in concentrations much greater than those of lithium interstitials under all considered synthesis conditions. The study proposed that supervalent doping could be an effective strategy to increase ionic conductivity in the system, especially under Li-poor synthesis conditions, while subvalent doping could have a detrimental effect on the RT ionic conductivity at low-to-moderate doping levels when compared to undoped Li_3_OCl.

As localized clustering can occur when extrinsic dopants and point defects interact with each other, and this phenomenon can have a detrimental effect on ionic mobility, one of the most relevant calculations when investigating doping effects is the evaluation of dopant-vacancy binding energies. For example, we studied the doping of Li_3_OCl with F^–^ and divalent cations (Mg^2+^, Ca^2+^, Sr^2+^, and Ba^2+^)^47^ via defect and MD simulations and found that while F-doped Li_3_OCl displayed high dopant-vacancy binding energies and low conductivity, its Mg-doped equivalent showed high ionic conductivity and low migration barriers with low dopant-vacancy binding energies. F doping was also predicted to cause high dopant-vacancy binding energies in Li-rich low-dimensional-networked antiperovskites.^6^ As a result, a significant reduction in Li-ion diffusion and an increase in activation energy were observed in F-doped Li_*x*_OA_*x*–2_ (A = Cl, Br; *x* = 3–6). This detrimental effect from F doping was attributed to its low polarizability compared to Cl and Br and its binding to Li vacancies when doped at the oxygen sites.

Doping of Na-based antiperovskites has also attracted significant attention. For example, Wan et al.^48^ used a combination of NEB and AIMD simulations to analyze Mg, Ca, Sr, Ba, and Ca doping in Na_3_OCl. The authors reported Ca^2+^ to be the most promising dopant tested for this material, as its addition leads to the lowest dopant-vacancy binding energy. More recently, we investigated the effects of divalent and trivalent dopants (Mg^2+^, Ca^2+^, Sr^2+^, Ba^2+^, Al^3+^, and Ga^3+^) on the ionic transport and conductivity in Na_3_OCl via large-scale atomistic calculations (Figure 4(c)–(e)).^49^ We found that alkali-halide Schottky defects are the dominant disorder in undoped Na_3_OCl and revealed Mg^2+^, Ca^2+^, Al^3+^, and Ga^3+^ as the most favorable dopants, with the smallest binding energy and highest conductivity displayed by the Mg-doped system (10^–5^ S cm^–1^ at 500 K). As shown in Figure 4(e), higher binding energies were found for Ca^2+^, Al^3+^, and Ga^3+^, indicating a significant level of vacancy/dopant clustering, resulting in reduced ionic conduction compared with that of their Mg-doped counterpart.

Due to the importance that effective ion migration has for the performance of solid-state batteries, it is unsurprising that a vast number of studies have also focused on promoting the understanding of the underlying mechanisms involved in ionic diffusion within solid electrolytes and uncovering the factors that can impact such mechanisms.

In the context of antiperovskites, mechanistic studies of ion transport represent a particularly interesting topic of discussion. This interest arises from many fundamental studies with sometimes conflicting theoretical predictions in recent years, especially regarding the dominance of interstitial or vacancy-mediated mechanisms. Emly et al. explored a three-ion hop mechanism that involved Li interstitial dumbbells in Li_3_OCl, Li_3_OBr, and Li_3_OCl_0.5_Br_0.5_ via DFT simulations.^50^ The authors found that such a mechanism leads to a very low Li-ion migration barrier of ∼0.17 eV for Li_3_OCl and Li_3_OBr, indicating a concerted Li-ion motion that is seen in several other solid electrolytes.^47,51−59^ However, the same report also found high formation energies for Li interstitials, indicating that the mechanism could not be responsible for the high conductivities observed experimentally.

Mouta et al.^60^ also attempted to elucidate the dominant ion transport mechanism in Li_3_OCl and found a low value of 0.13 eV for interstitial migration based on classical atomistic quasi-static calculations. However, as the concentration of Li vacancies found were 6 orders of magnitude greater than those for Li interstitials, the authors declared vacancy migration, with an energy barrier of 0.30 eV, to be the relevant ion transport mechanism in Li_3_OCl. Lithium vacancy hopping was also deemed to be the main ionic diffusion mechanism in Li_3_OCl by Lu et al.^61^ in a study that used classical MD and DFT simulations to investigate the defect chemistry and ionic transport of this material based on three types of charge neutral defect pairs (i.e., LiCl and Li_2_O Schottky pairs and a Li interstitial with an oxygen–chlorine antisite). The authors found that while all analyzed defect pairs have similar formation energies, they all lead to different ionic diffusivities. The LiCl-deficient Li_3_OCl displayed the highest ionic conductivity based on the formation of low energy Li transport pathways between the Cl vacancies.

The debate over whether the pertinent ion transport mechanism for antiperovskites is vacancy hopping or an interstitial mechanism was furthered by a classical atomistic quasi-static study by Mouta et al.^53^ that suggested that Li interstitials could become dominant in Li-rich antiperovskites when they are sufficiently Li-halide deficient. In addition, a blended perspective was offered by Stegmaier et al.,^51^ where Li vacancies will be present in Li_3_OCl near the cathode and Li interstitials will dominate near the anode–electrolyte interface. Vacancy hopping was found to be the preferred mechanism in our earlier work for a wide range of antiperovskites with the general formula Li_3–*x*_Na_*x*_OCl_1–*y*_Br_*y*_ (*x* = 1–2; *y* = 0–1).^27^ A vacancy-mediated mechanism was also reported to be the pertinent mechanism for Na_3_OCl by Wan et al.^48^ in a study that combined NEB and AIMD simulations. However, interstitials were shown to play a significant role in Ruddlesden–Popper antiperovskites with the general formula X_4_OA_2_ (X = Li or Na; A = Cl, Br, or I) in a study by Zhao et al.^54^ that compared vacancy and interstitials mechanisms, with the latter displaying lower migration barriers. In the context of Ruddlesden–Popper systems, using MD simulations, Jalem et al.^29^ revealed that the characteristic migration mechanism in inverse Ruddlesden–Popper tetragonal antiperovskites is fast Li-ion diffusion within the O–Li intraslab layer in the ab and ac/bc planes.

**Figure 5:**
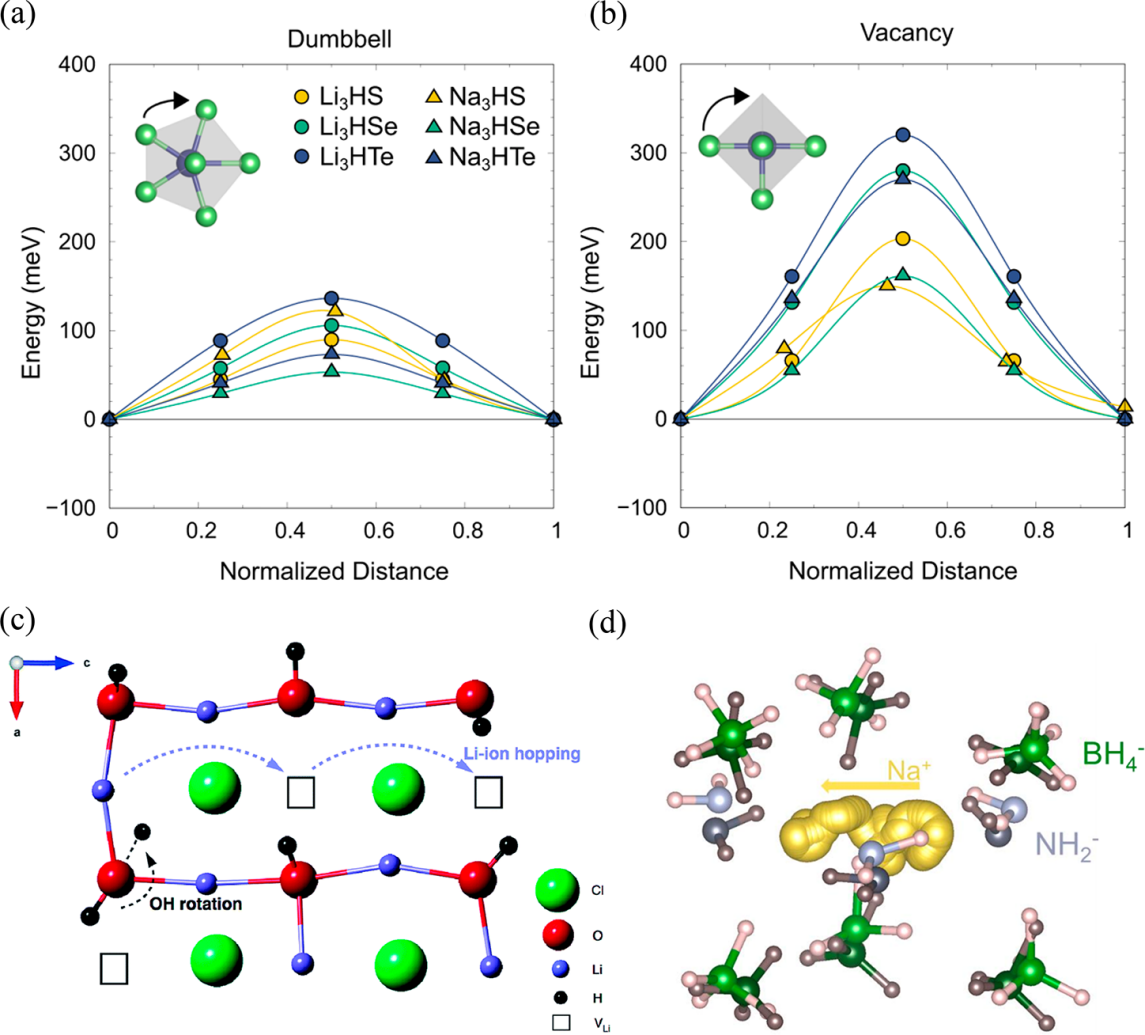
Low-barrier migration pathways for (a) vacancy and (b) interstitial dumbbell migration in M_3_HCh (M = Li or Na; Ch = S, Se, or Te) antiperovskites. Reproduced with permission from ref (62). Copyright 2021 The Authors. (c) Predicted Li-ion migration mechanism in Li_2_OHCl. Reproduced from ref (70) under a CC BY 3.0 license. (d) Atomic trajectories for a representative Na-ion migration event in Na_2_(NH_2_)(BH_4_) at 363 K. Every frame is shown for the Na ion, while for the neighboring cluster anions just the initial and final positions are shown (dark and light, respectively). Reproduced with permission from ref (73). Copyright 2020 Wiley-VCH.

More recently, Gao et al.^62^ successfully combined hydride anions (H^–^), endowed with large polarizability, and chalcogenide (Ch^2–^) anions to form a series of antiperovskites with soft anionic sublattices (M_3_HCh (M = Li or Na; Ch = S, Se, or Te)). The NEB-calculated energy barriers for the interstitial dumbbell hopping mechanism in these materials ranged from 0.05 to 0.14 eV (Figure 5(a)), values significantly lower than those calculated for vacancy-mediated mechanisms, which ranged from 0.15 to 0.32 eV (Figure 5(b)). These low migration barriers indicate a favorable dumbbell migration mechanism, because of the soft phonon mode associated with the rotational motion of the HM_6_ octahedra.

The impact of halide substitution on the ion transport properties of antiperovskites has also been considered. In our previous experimental–computational study,^63^ we used diffraction techniques, impedance spectroscopy, and AIMD simulations to explore mixed halide compositions with the formulas Na_3_OX (X = Cl, Br, I, and/or BH_4_), including Na_3_OCl_0.5_(BH_4_)_0.5_, Na_3_OBr_0.5_(BH_4_)_0.5_, Na_3_OI_0.5_(BH_4_)_0.5_, and Na_3_OCl_0.33_ Br_0.33_(BH_4_)_0.33_. We found a qualitative trend where increasing the halide size concomitantly increases conductivity and decreases the activation energy. This trend illustrates how the halide size impacts the cell volume and, thus, the Na–O distance. Longer Na–O distances lead to a weaker coordination and facilitated Na-ion hopping and, consequently, increased conductivity. Simultaneously, the impact of the halide size on the activation energy can be understood as the polarizability of the halogen controlling the activation energy for conduction via its effect on the lattice softness.^64,65^

Ion transport mechanisms in low-dimensional-networked antiperovskites have also been explored. In our recent study,^6^ the energetics of defect formation and Li-ion transport characteristics were investigated for a range of Li_*x*_OA_*x*–2_ (A = Cl or Br; *x* = 3–6) antiperovskites with zero- to three-dimensional-networked structures via atomistic simulations. The calculations revealed that for all systems analyzed, except for Li_4_OCl_6_, Li halide Schottky defect pairs are the dominant native defects, suggesting a preferred vacancy-mediated mechanism for the ionic diffusion in these systems. We obtained higher defect concentrations in the systems containing Cl as the halide but higher Li-ion diffusion for Br-based systems, illustrating the importance of lattice polarizability in these soft materials.

Interesting results regarding ionic transport were also obtained for the cluster-ion-based vacancy-rich Na_2_BH_4_NH_2_ material, which displayed a high ionic conductivity of 7.56 × 10^–4^ S cm^–1^ at 90 °C and an activation energy of 0.67 eV in an experimental–theoretical study by Jiang et al.^66^ The authors used AIMD simulations to investigate ionic migration and the role of vacancies in the system and revealed that Na ions could easily migrate within the bulk of Na_2_BH_4_NH_2_ via both interstitial and vacancy migration. Mean-squared displacement plots for this material showed that ionic diffusion was greater in Na vacancy-rich Na_2_BH_4_NH_2_ compared to that in Na vacancy-free Na_3_BH_4_NH_2_ and NaBH_4_ at 300 K, illustrating the positive impact vacancies have on ionic transport.

The effect of different structural features, such as polyanion rotation, on ionic migration has also been investigated. Fang et al.^21,30^ used DFT and AIMD calculations to explore the stability and ionic migration characteristics in superhalogen-based antiperovskites, including Li_3_OBH_4_, Li_3_SBF_4_, Li_3_OCl_0.5_(BH_4_)_0.5_, and Li_3_SCl_0.5_(BF_4_)_0.5_. The authors uncovered that the rotation and translation of the superhalogen clusters play a fundamental role in the enhancement of Li-ion migration in the analyzed systems, an effect often labeled the “paddlewheel effect”. The paddlewheel effect is often reported to be a pertinent factor for improved ionic migration in many antiperovskites, as well as in other solid electrolytes candidates.^2,55−57,67^ Song et al. used an array of materials characterization techniques and AIMD simulations to explore the ionic conductivities of Li_3–*x*_OH_*x*_Cl (*x* = 0–1) and Li_2_OHBr.^68^ Their results revealed an interesting mechanism for Li-ion transport, where low energy pathways for the formation of Frenkel defects and highly correlated Li-ion jumps were created by the rotation of the OH group. This mechanism results in fast and highly correlated ionic transport and was reported to significantly enhance the ionic conductivity of the analyzed systems. Similarly, in a DFT study that investigated the different phases of Li_2_OHCl, Howard et al.^69^ found that the presence of OH groups could impact the ionic motion in this hydrated system.

We also investigated the Li_3–*x*_OH_*x*_Cl system via integrated AIMD and solid-state NMR studies. As schematically presented in Figure 5(c), our results revealed a strong relationship between proton dynamics and long-range Li-ion transport based on Li-ion hopping and the rotation of the OH group, with Li-ion transport being highly correlated with proton concentration and Li-ion vacancy levels.^70^ Our atomistic simulations indicated fast Li-ion diffusion but ruled out the possibility of long-range proton diffusion due to the large separation between oxygen ions (∼4 Å). Similar conclusions were found by posterior works by Song et al.^71^ and Wang et al.^72^ Such studies indicate that the tailoring of proton content could be a useful strategy for optimizing the ionic conductivity of antiperovskite solid electrolytes.

More recently, the first instance of a double paddlewheel effect, where the rotational mobility of both anion groups promotes fast ionic migration, in an antiperovskite was reported by Tsai et al.^73^ in an experimental–theoretical study of Na_3–*x*_O_1–*x*_(NH_2_)_*x*_(BH_4_) (Figure 5(d)). The techniques used to confirm the concomitant importance of the rotation of both anion groups included AIMD, electrochemical impedance spectroscopy, powder and synchrotron X-ray diffraction, and NMR and neutron diffraction. The double paddlewheel effect leads to a Na-ion conductivity a factor of 10^2^ times higher at *x* = 1 compared to the result expected when only a vacancy-mediated mechanism is present. Although the study confirmed that the rotational activity of both anion groups is required to establish high ionic mobility, only the rotation of the amide group is synchronized with Na-ion migration with the mobility of the borohydride anions exhibiting an indirect and asymmetric relationship to the Na ions. Such an expansion of the paddlewheel effect concept and further investigation of this in other antiperovskites could accelerate their future discovery and design.

The use of computational design in probing ion transport mechanisms is particularly important, given that the experimental characterization of such phenomena can be challenging with significant barriers. These include the limited ability of many characterization methods to perform *in situ* monitoring, the need for expensive setups and hardware to carry out *in situ* measurements and the competitive nature of securing experimental time at national and international facilities to use techniques to probe ion migration, such as, for example, muon spin spectroscopy.^2^ Moreover, there are a plethora of factors that can impact ion migration in a system (e.g., composition, pellet texture, and thermal/physical history) that are often disregarded during characterization, which adds to the challenge of validating ionic migration experimentally.^2^ Nevertheless, atomistic modeling also faces a number of challenges and relies on significant assumptions when attempts are made to provide insights into the in situ performance of materials. In this context, numerous outstanding experimental–computational reports on the nature of ion transport in antiperovskite solid electrolytes have been published that overcome the inherent weaknesses of these two complementary paradigms, as described throughout this review.

More recently, ML has been employed to identify and quantify the relevance different structural, chemical, and physical features have on ionic mobility within antiperovskites. Kim and Siegel^74^ used 600 DFT-calculated hopping barriers to train ML algorithms to predict ion migration barriers in 36 antiperovskites with the general formula X_3_BA (X = Li, Na, or K; B = O, S, or Se; A = F, Cl, Br, or I). The authors used mean decrease in impurity and individual conditional expectation plots to quantify the importance of the tested features in the ionic migration displayed by the antiperovskite systems. The study revealed that lattice properties, such as channel width and hopping distance, are the features that have the greatest impact on cation migration, with migration barriers decreasing as the channel width increases and the hopping distance decreases. Such features were calculated to account for 50% of the total feature importance for interstitial migration and 70% of the importance for vacancy migration. Anion polarizability and defect formation energy were also considered significant features for ion mobility, comprising 22% for vacancy migration and 35% for interstitial migration, respectively. This study represents a significant advancement in the development and design of antiperovskite solid electrolytes, as it uncovers a subset of impactful features for ion mobility, with many of those being elementary and simple to evaluate. The work also opens up the exciting possibility of attempting to generalize the method for other families of solid electrolyte materials.

## GRAIN BOUNDARIES, SURFACES, AND ELECTROLYTE–ELECTRODE INTERFACES

Although the properties of individual materials are fundamental to device efficiency, the success of solid-state batteries also relies heavily on the interfaces within. However, as with many energy technologies, despite the importance of interfacial features, the current understanding around such topics in solid-state batteries is limited compared to that of bulk materials, which is largely due to the complexity involved in their investigation both experimentally and computationally. Nevertheless, recent years have seen a rise in the use of atomistic modeling in studying the structures, formation, and performance of interfaces in solid electrolytes and solid-state batteries.

The interfaces formed within a solid-state battery can be classified as heterogeneous (e.g., electrolyte–electrode interfaces) or microstructural (e.g., grain boundaries (GBs)).^2^ Given that both types of interfaces can greatly impact ionic conductivity and the overall device performance, understanding the influence and characteristics of such interfaces is critical.^2,3,75^ In this Review, we focus on highlighting the latest developments regarding the atomistic simulation of GBs, surfaces, and electrode–electrolyte interfaces.

We first consider GBs, which are defined as surfaces of contact between differently orientated crystallites that often display different structural and composition features when compared to the bulk crystal.^2^ GBs can significantly influence the overall conductivity of a material, either positively or detrimentally.^2,76−79^ Despite the fact that GBs are known to significantly impact the overall kinetics of ion transport, their pertinent properties and influence are still not fully understood, especially when compared to those of bulk materials.

To rectify this, we used large-scale MD simulations to explore ionic transport at a variety of representative low-energy GBs in Li_3_OCl.^23^ We predicted high GB concentrations and significant GB resistance in this material. We also proposed a polycrystalline model to quantitatively investigate the effect of GBs on conductivity as a function of grain size. This work draws attention to the importance of considering the impact of GBs on ionic conductivity when exploring solid electrolytes, because solely considering the bulk material can lead to a significant underestimation of the activation energy for ionic diffusion. This investigation has inspired subsequent computational works focused on GBs in Li_3_OCl^80,81^ and indeed other solid electrolytes. For example, Chen et al.^80^ used DFT to investigate the behavior of GBs in Li_3_OCl and also predicted high concentrations of GBs and GB resistance, aligned with our previous results.^23^

More recently, Shen et al.^81^ used a combination of first-principles calculations and phase field modeling to quantify the impact of GBs on ionic conduction in Li_3_OCl. The authors considered the interaction between point defects and GBs in their calculations at different scales, offering insight into point defect segregation at the GBs. Usually, the high ionic conductivity displayed by solid electrolytes is heavily reliant on the concentration of point defects in the lattice. As GBs are considered favorable sites for the segregation of point defects, it is natural to deduce that GB segregation could affect the distribution of point defects in the lattice and, consequently, impact its ionic conductivity. The calculations by Shen et al. showed that defect segregation varies with GB orientation, with Li-vacancy segregation energies lowering with the enhancement of GB coherency. This work also showed that defect segregation strengthens the detrimental impact of GBs on ionic conduction by approximately 1 order of magnitude. However, the authors reported that such an impact is not significant for grain sizes of hundreds of nanometers, suggesting that ionic conductivity could be improved by tuning the structure and grain size of the GBs.

In a combined experimental–theoretical study, we explored the role of GB resistance in affecting the total Li-ion conductivity in a range of hydrated antiperovskites with the formula Li_2_OHCl_1–*x*_Br_*x*_ (*x* = 0.0, 0.1, 0.3, 0.5, 0.7, 0.9 or 1.0).^82^ Using DFT simulations, we were able to determine the Li-ion diffusion coefficients at the grains and GBs of the materials, with significantly reduced diffusion found for the latter. This was experimentally corroborated using electrochemical impedance spectroscopy on pellets with a controlled grain size.

**Figure 6:**
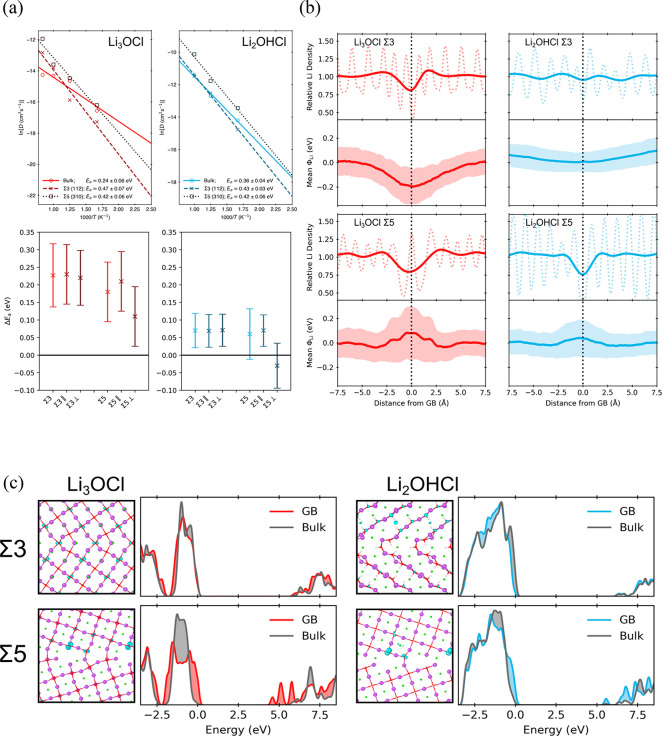
(a) Li-ion diffusivities for the bulk and GBs of Li_3_OCl and Li_2_OHCl. Relative activation energies in the GBs compared to bulk associated with the total diffusion and decomposed into components parallel (∥) and perpendicular (⊥) to the GB plane are shown below each diffusivity plot. (b) Relative densities of Li (top panels) and mean electrostatic potentials around Li ions, φLi (bottom panels), as a function of distance from the GB for Li_3_OCl and Li_2_OHCl at 600 K. (c) Projected density of states with associated GB structures for Li_3_OCl and Li_2_OHCl. Energies are referenced against the position of the valence band maximum in the bulk-like region. Partial charge density isosurfaces show the highest occupied orbital (turquoise). Reproduced with permission from ref (83). Copyright 2023 The Authors.

More recently, we utilized first-principles calculations to establish design principles for GBs in four promising solid electrolyte candidates, namely, Li_3_OCl, Li_2_OHCl, β-Li_3_PS_4_, and Li_3_InCl_6_.^83^ As displayed in Figure 6(a), our results show that the GBs in Li_3_OCl exhibit large barriers to ionic conductivity, while those in its hydrated material are less severe. This could be understood as a consequence of the perturbation of the electrostatic potential by the GBs in this material not being as significant (Figure 6(b)). The addition of highly polarizable ions or those that can adapt to electric fields by reorientation could mitigate the negative effects of electrostatic perturbations. Furthermore, our simulations showed that even when GBs do not significantly affect ionic conductivity, they can still disrupt the electronic structure and lead to undesirable electrical conductivity and potential lithium dendrite propagation (Figure 6(c)). We also showed for the first time how correlated motion (e.g., the paddlewheel mechanism) can vary significantly at the GBs compared with the bulk.

We now shift our attention to the latest developments in the computational exploration of antiperovskite solid electrolyte–electrode interfaces, which are essential in determining the stability and performance of solid-state batteries at the device scale. Similar to GB investigations, the complexities involved in explicitly modeling electrolyte–electrode interfaces have led to a relative scarcity of such explorations. Nevertheless, recent progress in this field for antiperovskites has been made and resulted in an enhanced understanding of their interfacial phenomena.

**Figure 7:**
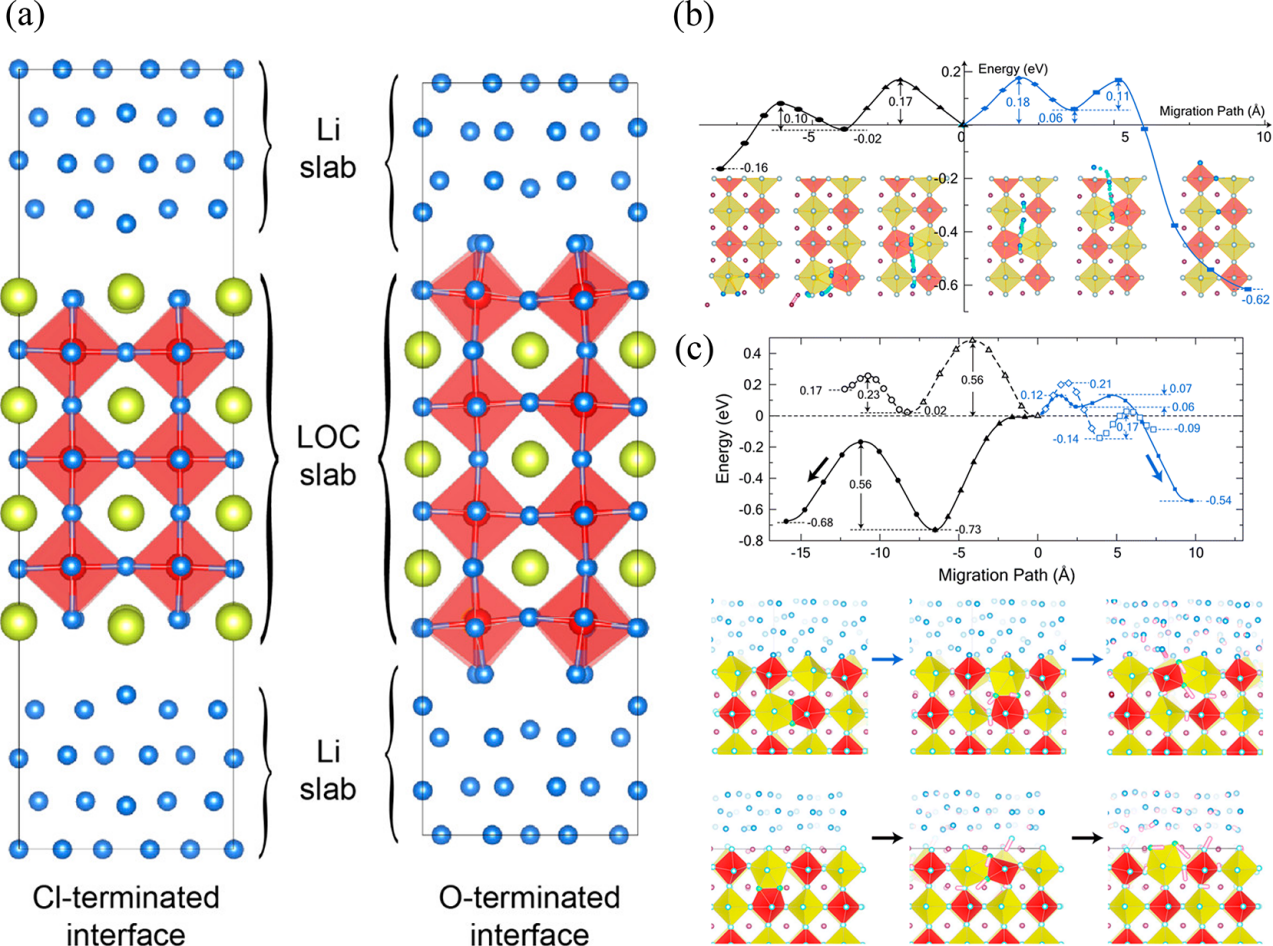
(a) Relaxed structures of Cl- and O-terminated interfaces consisting of seven layers of Li_3_OCl (100) and bcc Li (100) planes. Reproduced with permission from ref (84). Copyright 2019 American Chemical Society. Energy profiles for the migration of a Na interstitial (b) toward Na–I and Na–S–O surface terminations along the path shown in the bottom panel for snapshots during migration and (c) near Na_6_SOI_2_/Na (001) interfaces. Black (blue) curve in the top-left (top-right) panel represents the migration of a Na interstitial from an inner bulk site toward the Na–I (Na–S–O) surface and Na–I/Na (001) (Na–S–O/Na (001)) interface. Reproduced with permission from ref (89). Copyright 2023 Royal Society of Chemistry.

Prior to exploring such interfaces explicitly using atomistic methods, it is necessary to determine which electrolyte surfaces are the most stable for the system of interest. Kim and Siegel^84^ used DFT calculations to investigate the stability of a selection of low-index nonstoichiometric surfaces of Li_3_OCl with different terminations. Their simulations revealed that the LiCl-terminated (100) surface displayed the lowest surface energy of 0.19 J m^–2^ (at 300 K), suggesting that this surface is the most likely to form Li_3_OCl at equilibrium. Using these surfaces, the authors investigated the electronic and thermodynamic properties of the interface between Li_3_OCl and a Li metal anode on the atomic scale (Figure 7(a)). The authors computed a plethora of properties, including the work of adhesion, electrochemical windows, interfacial energy, wettability, and band edge shifts. Their calculations revealed the oxygen-terminated interface as the most thermodynamically stable interface, and the large work of adhesion found suggests that Li will wet Li_3_OCl, indicating potentially low interfacial resistance. Nevertheless, the simulations showed that this interfacial interaction also reduced the electrochemical window of Li_3_OCl, suggesting that there is a trade-off between strong interfacial bonding and electrochemical stability. Despite the reduction in the electrochemical window, it is noteworthy that the conduction band minimum remained ∼1 V more negative than the Li/Li^+^ redox potential, suggesting stability against reduction by the anode.

A later study performed by Wu et al.^85^ also found the Li–Cl-terminated (100) surfaces to be the most favorable in Li_3_OCl. The interface between Li_3_OCl and a Li metal anode was then studied by these authors via first-principles and AIMD calculations considering its interfacial charge distribution, geometric structure, electronic properties, and structural stability. They found that the interface was stable at 0 K and operating temperatures. Their AIMD calculations revealed that the Li-ion migration in their model was predominantly along the interface boundary. The calculated self-diffusion, conductivity, and activation energy of Li ions in the interface at 300 K were found to be 0.88 × 10^–5^ cm^2^ s^–1^, 1.60 S cm^–1^, and 0.09 eV, respectively. These values were greater than those for their counterparts in the bulk Li_3_OCl bulk, suggesting that this interface positively contributes to ionic transport.

The interface between Li_3_OCl and an almost ideal metallic intercalation cathode was explored by Stegmaier et al.,^51^ in a DFT study that used a polarizable continuum model. The calculations showed that high Li vacancy concentrations will build up in a single layer of the electrolyte at the interface with the cathode, forming a compact double layer. The onset of oxidation for Li_3_OCl and its subsequent products was investigated using first-principles simulations by Emly et al.^50^ The authors calculated the onset of oxidation for Li_3_OCl at 2.55 V relative to Li metal and predicted the formation of Li_2_O_2_ and LiCl. Richards et al.^86^ also carried out oxidation analysis of this material and predicted the onset at 3.00 V relative to Li metal with LiCl, ClO_3_, and LiClO_3_ as products. As such products are electronic insulators, it is expected that a passivated interphase can be formed at high voltages, which could protect the system from further oxidation.^2,87^ Similar oxidation explorations have also been conducted for Na-based antiperovskites. Lacivita et al.^88^ found an oxidation limit of 1.79 V relative to Na metal for Na_3_OBr with anodic reaction products Na_2_O_2_ and NaBr and an oxidation limit of 1.66 V relative to Na metal for Na_4_OI_2_ with NaI and NaIO_3_ as anodic reaction products.

More recently, a first-principles study performed by Choe et al.^89^ focused on the interface between a metallic Na anode and the Na-based antiperovskite Na_6_SOI_2_. Their calculations showed that the Na_6_SOI_2_ (001) surface with two different terminations (i.e., Na–S–O and Na–I) could be used along with the Na (001) and (101) surfaces to form four different interface models due to their calculated exothermic interlayer binding energies, which ranged from −18.3 to −15.8 meV Å^–2^, and formation energies of −28.0 to −21.5 meV Å^–2^. The study analyzed Na-ion conductivity at these interfaces and found that interstitial Na–Na dumbbell migration could be the reason for the ionic conductivity in the system due to a low calculated activation energy of ∼0.17 eV inside the electrolyte. The study also revealed that ionic migration toward the anode is favored by the interstitial mechanism, while the reverse path is favored by a Na vacancy-mediated mechanism (Figure 7(b),(c)).

## CONCLUSIONS AND OUTLOOK

In this Review, we have highlighted some of the exciting latest developments in the computational design of antiperovskite solid electrolytes as a highly promising material family whose literature has experienced exponential growth in recent years. We have discussed the progress to date regarding the discovery and screening of new antiperovskite systems and emphasized the importance of multitargeted studies of properties of interest (e.g., ionic conductivity, lattice distortion, thermodynamic stability, electrochemical stability, synthesizability), especially when said properties compromise or contradict one another. The novel correlations between properties found for antiperovskites have revealed, for example, the discovery that larger lattice distortions provide lower energy barriers for ion migration and stabilities and higher synthesis temperatures, suggesting a 3-fold mobility–stability–synthesis trade-off. We have commented on the widely accepted unsuitability of the traditional Goldschmidt tolerance factor for describing antiperovskite systems, especially those containing heavy halides, and the recent report of a modified Goldschmidt tolerance factor as a successful descriptor for thermodynamic stability and band gap energy for antiperovskites with Ruddlesden–Popper structures.

High-throughput screening and ML-assisted methods will be essential for the timely discovery and further development of antiperovskite solid electrolytes. Herein, we have drawn attention to the latest reports in this field, including a general graph neural network model capable of assessing the synthesizability of antiperovskites and a ML-assisted method that can accelerate the evaluation of kinetic properties in antiperovskites by removing a critical bottleneck of such evaluations, namely, the use of time-consuming NEB calculations. Such works naturally raise the question of whether (or when) similar methodologies can be applied and obtained for more exotic antiperovskites, for example, multivalent cation (e.g., Mg^2+^ or Ca^2+^)-based and low-dimensional-networked antiperovskites and structures with significant octahedral tilting.

In this context, antiperovskites based on chemistries beyond Li and Na ions, such as systems containing mobile K, Mg, or Ca ions, also offer an intriguing research path. Due to their wide availability, such chemistries are becoming increasingly appealing to battery applications. In fact, a number of atomistic studies have already been published in this area, with compositions such as K_3_OI, K_2.9_Ba_0.05_OI,^90^ Mg_3_NAs, Ca_3_NAs, and Ca_3_PSb^91^ providing interesting results. Such promising reports represent a new avenue for future investigations of high-performance antiperovskites with more abundant elements for a wider range of energy storage applications.

In recent years, several different doping strategies that can have a significant impact on the ionic conductivity of antiperovskites have also attracted interest. Herein, we have discussed recent findings for doped Li- and Na-rich antiperovskite systems, with the majority of these suggesting a detrimental effect on ionic diffusion caused by fluorine doping. In contrast, the doping of antiperovskite systems with divalent ions, especially Mg^2+^ and Ca^2+^, has shown promising results (i.e., high ionic conductivity and/or low dopant-vacancy binding energies), highlighting a promising future direction for compositional design.

The ongoing debate regarding the domination of interstitial- or vacancy-mediated mechanisms in antiperovskite systems has also been covered. Conflicting ideas have been proposed, and this trend continues with the debate not yet resolved. Interesting results focused on ion transport mechanisms in antiperovskites have also been presented, with intriguing reports determining the correlations between lattice distortion and the barriers for ion migration. We have drawn attention to the importance of the paddlewheel effect in antiperovskite solid electrolytes, including the first report of a double paddlewheel effect, where the rotational mobility of two anion groups promotes fast ion migration. It can be anticipated that such novel expansion of the paddle-wheel effect concept and further investigation of this in other antiperovskite systems could greatly accelerate their future discovery and design. Furthermore, the exploration of ionic conductivity and transport mechanisms in solid electrolytes under the influence of stack pressures, which are typically required for practical SSBs, has so far been limited to only a few computational studies. This represents an important area of future work for antiperovskite solid electrolytes and fast ion conductors generally.

We have also discussed the latest developments in the application of ML algorithms to predict ion migration barriers in antiperovskite systems, with lattice properties, such as channel width and hopping distance, being found to be the features that have the greatest impact on cation migration. Such explorations represent a great leap forward in the design of antiperovskite solid electrolytes as they uncover a subset of impactful features for ion mobility, with many of those being elementary and straightforward to determine.

Despite the recent rise in the number of studies focusing on interfacial explorations, this Review stresses how the complexity involved in computationally investigating interfacial phenomena and features has hindered advances in the field. Nonetheless, recent results have provided critical knowledge around pertinent properties and mechanisms. We illustrate the recent progress in the area by discussing works that collectively suggest that the detrimental impact of GBs on ionic conduction can be reduced by tuning the structure and grain size of the atomistic GBs, with antiperovskites with larger grain sizes and coherent GBs expected to display enhanced Li-ion transport. We also comment on recent findings that point to the existence of a trade-off between strong interfacial bonding and electrochemical stability between Li_3_OCl and Li metal anodes.

We have illustrated how transformative ML-assisted investigations are anticipated to be in the field of interfacial exploration, an area that still severely suffers from the complexities associated with accurately simulating the interfaces they focus on. To the best of our knowledge, no ML-based study has focused on exploring the interface between electrodes and antiperovskite solid electrolytes.

As seen in the examples discussed in this Review, the current primary computational methods of choice for investigating interfaces in solid-state batteries at the atomistic scale are based on either DFT or classical force field-based methods. Nevertheless, the use of ML force fields for interfacial explorations is widely expected^92−94^ to enable the DFT-accurate treatment of much more complex and larger simulation cells and time scales, which will be key for investigating interfacial problems, such as sources of charge accumulation and mass transfer resistance at the interfaces (e.g., GBs, chemical and electrochemical reactions and decomposition products) and allow for the design of stable conducting interfaces in which practical solid-state batteries can be built on.

Although progress has been recently made regarding the creation and exploitation of explicit atomistic models, there are still many unanswered questions. Furthermore, most studies have only focused on Li_3_OCl and its hydrated form, leaving the understanding regarding other antiperovskite compositions unexplored. Nevertheless, these studies clearly demonstrate the potential of such simulations in both understanding and designing new antiperovskite solid electrolytes with good performance in their bulk and at their interfaces.
